# Increasing nausea and vomiting of pregnancy is associated with sex-dependent differences in early childhood growth: the GUSTO mother-offspring cohort study

**DOI:** 10.1186/s12884-021-04024-9

**Published:** 2021-08-22

**Authors:** Judith Ong, Suresh Anand Sadananthan, Shu-E Soh, Sharon Ng, Wen Lun Yuan, Izzuddin M Aris, Mya Thway Tint, Navin Michael, See Ling Loy, Kok Hian Tan, Keith M Godfrey, Lynette P Shek, Fabian Yap, Yung Seng Lee, Yap Seng Chong, Shiao-Yng Chan

**Affiliations:** 1 Department of Obstetrics & Gynaecology, Yong Loo Lin School of Medicine, National University of Singapore, 1E Kent Ridge Road, NUHS Tower Block Level 12, Singapore, 119228 Singapore; 2 Singapore Institute for Clinical Sciences, A*STAR, Singapore, Singapore; 3 Department of Paediatrics, Yong Loo Lin School of Medicine, National University of Singapore, Singapore, Singapore; 4 Department of Population Medicine, Division of Chronic Disease Research Across the Lifecourse, Harvard Medical School and Harvard Pilgrim Health Care Institute, Boston, USA; 5 Department of Reproductive Medicine, KK Women’s and Children’s Hospital, Singapore, Singapore; 6 Duke-NUS Medical School, Singapore, Singapore; 7 Department of Maternal Fetal Medicine, KK Women’s and Children’s Hospital, Singapore, Singapore; 8 MRC Lifecourse Epidemiology Unit & NIHR Southampton Biomedical Research Centre, University of Southampton & University Hospital Southampton NHS Foundation Trust, Southampton, UK; 9 Lee Kong Chian School of Medicine, Nanyang Technological University, Singapore, Singapore; 10 Department of Paediatric Endocrinology, KK Women’s and Children’s Hospital, Singapore, Singapore

**Keywords:** Hyperemesis gravidarum, Premature birth, Child anthropometry, Child growth

## Abstract

**Background:**

Nausea and vomiting of pregnancy (NVP) is common and underlying mechanisms are poorly understood. Longer-term offspring outcomes are also not well documented. This study aimed to determine if NVP, even in milder forms, is associated with adverse pregnancy and childhood growth outcomes.

**Methods:**

In the GUSTO prospective mother-offspring cohort, women with singleton pregnancies (*n* = 1172) recruited in first trimester responded to interviewer-administered questions at 26–28 weeks’ gestation about earlier episodes of NVP since becoming pregnant. Pregnancy outcomes were obtained from medical records. Offspring height and weight measured at 15 time-points between birth to 72 months (m) were standardised for age and sex.

**Results:**

58.5% (*n* = 686) reported mild-moderate vomiting (mNVP), 10.5% (*n* = 123) severe vomiting (sNVP) and 5.7% (*n* = 67) severe vomiting with hospitalisation (shNVP). There was no difference in odds of gestational diabetes, hypertensive disorders of pregnancy, labour induction or caesarean section after adjustment for covariates. sNVP was associated with late preterm delivery [34^+ 0^–36^+ 6^ weeks’, adjusted OR = 3.04 (95% CI 1.39,6.68)], without increased odds of neonatal unit admission. Compared with no NVP, boys born to mothers with sNVP were longer at birth [adjusted β = 0.38 standard deviations (SDs) (95% CI 0.02,0.73)], remained taller [0.64 SDs (0.23,1.04) at 72 m] and heavier [0.57 SDs (0.05,1.08) at 60 m] without differences in BMI. Conversely, girls born to mothers with shNVP were lighter from 48 m [− 0.52 SDs (− 1.00, − 0.03)] onwards with lower BMI [− 0.61 SDs (− 1.12,-0.09)]. Conditional growth modelling revealed significant sex-divergence in weight-gain at birth-3 m, 6-9 m and 4–5 years.

**Conclusions:**

Severe NVP was associated with late preterm delivery, and both mild-moderate and severe NVP associated with sex-dependent differences in early childhood growth. Boys whose mothers had NVP were taller and heavier from birth with faster growth in the first year, whereas, girls had poorer weight gain and were lighter by 48 m. As even milder severities of NVP could have long-term impact on offspring growth, further research is needed to determine mechanisms involved and implications on future health.

**Trial registration:**

Clinicaltrials.gov identifier NCT01174875.

## Background

Nausea and vomiting of pregnancy (NVP) is common in early pregnancy. Symptoms range from mild nausea to severe vomiting, also known as hyperemesis gravidarum. Its reported incidence varies depending on definition criteria and population. One study from Canada reported an NVP incidence of 63% [1], whilst another in a Chinese population found an incidence as high as 91% [2]. Symptoms typically begin in the first trimester and continue until 14–16 weeks’ gestation.

Whether NVP has any adverse effects on pregnancy, fetal and childhood outcomes have long been debated. In meta-analysis of retrospective studies and population-based studies, there have been small increased risks of preterm birth (odds ratio (OR:1.18–1.32) and small-for-gestational-age neonates (OR:1.06–1.32) associated with the most extreme or severe NVP manifestation of hyperemesis gravidarum [2]. Hyperemesis gravidarum is characterized by at least 5% weight loss compared to pre-pregnancy weight, dehydration and electrolyte imbalances [3–5], which is postulated to be largely due to changes in the quality and quantity of maternal dietary intake [6, 7]. Increased pregnancy adversity was reported mainly amongst those with hyperemesis gravidarum extending into the second trimester [8].

As existing studies have focussed predominantly on severe NVP [3–5], it is unclear if milder NVP, without apparent weight loss, dehydration or significant change in maternal oral intake, is of clinical significance on pregnancy and offspring health. Although it is a commonly held view that the adversity from NVP is due to poor maternal intake, the underlying mechanisms are poorly understood and may include poor psycho-emotional health, variations in genetic polymorphisms and other factors [9], which may not correlate with the severity of NVP but could influence pregnancy and offspring development. This is important as majority of pregnant women have mild self-limiting symptoms [8] and are often minimally treated. If milder NVP is associated with adversity, the approach to managing this common pregnancy symptom should change if causality is proven.

In addition, long-term outcomes in offspring of women with NVP have rarely been investigated. In the context of childhood growth and early life metabolic programming, sex-dependent differences are also frequently reported [10–12]. Furthermore, the conception of girls has been associated with a higher risk of NVP than conception of boys [13]. Thus, the role of sex needs to be considered in investigations on offspring outcomes.

We hypothesised that there is a gradation of effect resulting in a higher risk of adverse pregnancy outcomes and aberrant childhood growth with increasing severity of NVP.

This study categorised pregnant women into four different groups according to the severity of NVP. We sought to describe differences in the NVP severity groupings linked with sociodemographic characteristics of women. Next, we aimed to assess if increasing severity of NVP was associated with pregnancy complications and differences in early childhood auxology separately in boys and girls.

## Methods

The Growing Up in Singapore Towards healthy Outcomes (GUSTO) mother-offspring cohort is a prospective study aimed at evaluating the influence of inherited and environmental exposures during early development, on pathways to metabolic compromise, altered body composition and future non-communicable diseases [14]. The National Healthcare Group Domain Specific Review Board and the SingHealth Centralized Institutional Review Board ethically approved this study. Informed written consent was obtained from each participant.

Pregnant women aged 18 years and above were recruited in the first trimester from Singapore’s two major public maternity units, KK Women’s and Children’s Hospital and National University Hospital between June 2009 and September 2010 (*n* = 1247). Participants and their children had homogenous parental ethnic background (Chinese, Malay or Indian).

At 26–28 weeks’ gestation, women with singleton pregnancies responded to structured interviewer-administered questionnaires about earlier episodes of nausea and vomiting since becoming pregnant. Using these self-reported responses of retrospective recall alongside verification from hospitalisation data in medical records, women were classified into 4 groups: (i) no vomiting; (ii) mild-moderate vomiting (mNVP), which included women who had “nausea only” and those who had “vomiting occasionally”; (iii) severe vomiting (sNVP), defined as “regular vomiting with inability to retain meals”; and (iv) severe vomiting with hospitalisation (shNVP), defined as vomiting requiring admission for intravenous rehydration. Data on age, ethnicity, educational attainment, parity and maternal smoking were prospectively collected through questionnaires at the same time.

Self-reported pre-pregnancy weight, and measured weights in clinics taken nearest to 14, 20 and 34 weeks’ gestation were recorded. Height was measured with a portable stadiometer (Seca 213, Hamburg, Germany) at 26–28 weeks’ gestation. Body mass index (BMI) was determined using the formula of weight (kg)/ height (m^2^). The strong correlation (*r* = 0.96, *p* < 0.001) between pre-pregnancy BMI and early pregnancy BMI at the first clinic visit provided confidence in self-reported pre-pregnancy weights, which was used in the calculation of gestational weight gain (GWG), defined as weight change from pre-conception until each of the gestational time points reported above.

Pregnancy and neonatal outcomes were extracted from medical records. Gestational diabetes mellitus (GDM) screening was universal and was defined using the WHO 1999 criteria in use at that time [75 g oral glucose tolerance test with elevated fasting (≥7.0 mmol/L) and/or 2 h (≥7.8 mmol/L) glucose measures]. Hypertensive disorders of pregnancy included pre-eclampsia and pregnancy-induced hypertension defined as new onset of blood pressure (BP) ≥140/90 mmHg on at least two determinations 4 h apart occurring after 20 weeks’ gestation, with pre-eclampsia cases also displaying proteinuria ≥300 mg/24 h or a dipstick reading of ≥1+ or abnormal liver function or elevated uric acid, as well as pre-eclampsia super-imposed on chronic hypertension. Caesarean deliveries included both elective and emergency cases. Induced labour included the use of prostaglandins or oxytoxin for labour onset. Preterm delivery was defined as births before 37 completed weeks’ gestation based on first trimester crown-rump length ultrasonographic measurements. Preterm births were subdivided into early (< 34 weeks) and late (34^+ 0^–36^+ 6^ weeks) groups. Infant birthweight was obtained from medical records. Small-for-gestational-age (SGA; <10th percentile) and large-for-gestational-age (LGA; >90th percentile) were identified using cohort-specific birthweight centiles customised for sex and gestational age according to Mikolajczyk et al. [15]. Neonatal unit (NNU) admissions included intensive and other lower levels of care.

Child weight and length/height were measured using standardised protocols [16] and BMI (in kg/m^2^) derived. Recumbent infant length at birth, 3 weeks, 3, 6, 9, 12, 15, 18, 24 months (m) and standing height at 36, 48, 54, 60, 66, 72 m were measured to the nearest 0.1 cm using an infant mat (SECA 210 Mobile Measuring Mat; SECA Corp.) or SECA 213 Stadiometer accordingly. Child weight was measured to the nearest gram using a calibrated scale (SECA 334 Weighing Scale) up to 18 m, and SECA 803 Weighing Scale from 24 m onwards. All measurements were taken in duplicate and averaged. Age- and sex- specific z-scores for weight, length/height and BMI were calculated as described in the WHO Child Growth Standards 2006 [17].

Conditional weight or height/length gain was computed as the standardised residual of current weight regressed on all previous weight or height/length measures [18]. It denotes how much the child deviates from his or her expected weight/height, given his or her prior measurements. For example, a positive conditional weight gain at age 3–4 years indicates that the child experienced a faster weight gain during this interval than expected for his/her current weight and previous weights. The conditional weight/height gain measures at different intervals are not correlated to each other. We determined the conditional weight/height gain at annual intervals between birth and 6 years, and also at 3-monthly intervals in the first year of life during the period of rapid growth.

Statistical analyses were performed with SPSS 24.0 and SAS (v9.4) software. Univariate analysis was done to compare baseline characteristics between NVP groups. Multivariate analyses were performed using multinomial logistic or linear regression to calculate odds ratios (OR) and beta coefficients (β) with 95% confidence intervals (CI), with adjustment for relevant covariates. Pregnancy and neonatal outcomes were adjusted for factors that were different between NVP groups [ethnicity, parity, sex (for pregnancy outcomes only)] and for variables previously found to influence pregnancy and child growth outcomes in the GUSTO cohort [maternal education, maternal age (continuous), parity (nulliparous or parous), maternal pre-pregnancy BMI (continuous), and maternal smoking (non-smoker or smoker, defined as self-reported smoker or plasma cotinine level above the detection limit of 0.17 ng/ml at 26 weeks’ gestation) [19–22]. A priori, child outcomes were stratified by sex for analyses and similarly adjusted for covariates (as listed at the bottom of tables) since sex is a potential confounder, and such an approach will be less susceptible to bias. As further affirmation of this approach, the effect modification by child sex in the association between NVP and conditional weight/height/length gain was tested by adding a multiplicative interaction term ‘NVP x sex’ as an independent variable in the model. Statistical significance was set at *p* < 0.05.

The GUSTO study is funded by the Singapore National Research Foundation under its Translational and Clinical Research Flagship Programme administered by the Singapore National Medical Research Council (NMRC/TCR/004-NUS/2008; NMRC/TCR/012-NUHS/2014). Additional funding is provided by the Singapore government-funded Agency for Science Technology and Research (A*STAR). The funders played no role in the research conduct and writing of this paper.

## Results

A total of 1172 women (94% of recruited) responded to the NVP questionnaire, of whom 25.3% (*n* = 296) of women reported no NVP, 58.5% (*n* = 686) reported mNVP, 10.5% (*n* = 123) sNVP and 5.7% (*n* = 67) shNVP. Of the 686 that were in the mNVP group, 336 (28.7% of 1172) had nausea only and 350 (29.9% of 1172) reported occasional vomiting.

Examination of sociodemographic characteristics linked with NVP found that the factors of ethnicity, parity and child sex were associated with severity of NVP (Table 1). Compared with Chinese women, Malay [unadjusted OR (uOR) 2.69 (1.39–5.21), *p* = 0.003] and Indian women [uOR 3.45 (1.79–6.66), *p* = 0.0002] were more likely to experience shNVP. Being parous compared with being nulliparous was also associated with increased likelihood of experiencing sNVP [uOR 1.59 (1.03–2.45), *p* = 0.037] and shNVP [uOR 1.98 (1.14–3.45), *p* = 0.016]. Women carrying girls were more likely to experience sNVP [uOR 1.62 (1.05–2.49), *p* = 0.029] than those carrying boys. There were no differences in NVP groups with respect to maternal age, pre-pregnancy BMI, maternal height, education levels and maternal smoking.

**Table 1:** Comparison of participant characteristics between groups classified by severity of nausea and vomiting of pregnancy

	**No vomiting** **(*n*** **= 296)**		**Mild-Moderate vomiting (*n*** **= 686)**		**Severe vomiting** **(*n*** **= 123)**		**Severe vomiting with hospitalisation (*n*** **= 67)**		***p*- value**
***Maternal***									
**Age at delivery,** years (median, IQR)	31.2 (27.8, 35.2)		31.1 (27.5, 34.9)		31.6 (27.6, 34.7)		30.3 (26.4, 34.3)		0.59
**Ethnicity** (*n*, %)									
Chinese (*n* = 662)	172 (26.0%)		399 (60.3%)		70 (10.6%)		21 (3.2%)		< 0.001*
Malay (*n* = 298)	67 (22.5%)		182 (61.1%)		27 (9.1%)		22 (7.4%)		
Indian (*n* = 212)	57 (26.9%)		105 (49.5%)		26 (12.3%)		24 (11.3%)		
**Pre-pregnancy BMI,** kg/m^2^ (median, IQR)	22.0 (19.6, 25.1)		21.3 (19.4, 24.8)		21.5 (19.4, 24.9)		22.6 (20.4, 26.5)		0.10
**Maternal Height,** cm (mean, SD)	158.3 (5.3)		158.3 (5.6)		157.6 (5.9)		158.5 (5.7)		0.82
**Parity** (*n*, %)									
**Nulliparous** (*n* = 544)	153 (28.1%)		317 (58.3%)		51 (9.4%)		23 (4.2%)		
**Parous** (*n* = 628)	143 (22.8%)		369 (58.8%)		72 (11.5%)		44 (7.0%)		0.03*
**Education level** (*n*, %)									
University (*n* = 396)	100 (25.3)		245 (61.9)		37 (9.3)		14 (3.5)		0.27
Post-secondary/Pre-university (*n* = 291)	69 (23.7)		170 (58.4)		32 (11.0)		20 (6.9)		
Secondary or below (*n* = 470)	125 (26.6)		261 (55.5)		52 (11.1)		32 (6.8)		
**Maternal smoking**^**#**^ (*n*, %)	42 (23.3)		110 (61.1)		15 (8.3)		13 (7.2)		0.50
***Child sex (n, %)***									
**Boys** (*n* = 609)	157 (25.8%)		374 (61.4%)		50 (8.2%)		28 (4.6%)		0.014*
**Girls** (*n* = 563)	139 (24.7%)		312 (55.4%)		73 (13.0%)		39 (6.9%)		
***Weight change from preconception (kg)***									
	**median (IQR)**	**β** **(95%CI)**	**median (IQR)**	**β** **(95% CI)**	**median (IQR)**	**β** **(95% CI)**	**median (IQR)**	**β** **(95% CI)**	
14 weeks’ gestation	2.65 (0.90, 4.75)	Ref	2.15 (0.57, 4.12)	−0.34 (−0.99, 0.30)	1.20 (0.55, 3.50)	−0.90 (−1.89, 0.08)	− 0.40 (−2.50, 2.10)	−2.80 *(− 4.17, −1.43)	
20 weeks’ gestation	5.95 (3.90, 8.17)	Ref	5.20 (3.40, 7.50)	− 0.40 (− 1.01, 0.19)	4.20 (2.20, 6.60)	−1.33 *(− 2.30, − 0.36)	4.40 (0.70, 5.55)	−2.39 *(−3.72, − 1.07)	
34 weeks’ gestation	11.60 (8.97, 14.85)	Ref	11.35 (8.60, 14.37)	−0.23 (− 0.98, 0.52)	10.60 (7.90, 13.80)	0.37 (− 1.55, 0.80)	10.20 (6.52, 14.47)	−1.61 *(− 3.16, − 0.05)	

*p-value < 0.05. ^**#**^self-reported smoker or plasma cotinine level above the detection limit ≥0.17 ng/ml at 26 weeks’ gestation

All **β** adjusted for ethnicity, maternal education, maternal age, parity, pre-pregnancy BMI, maternal smoking and offspring sex

To validate our classification of NVP we examined maternal weight changes from preconception and into pregnancy (Table 1) and confirmed that with increasing severity of vomiting, there was lessening gestational weight gain at 14, 20 and 34 weeks’ gestation, as expected. After adjusting for covariates, those with shNVP had the least weight gain at 14 weeks’ and at 20 weeks’ gestation compared with women with no NVP, while women with sNVP only showed less weight gain by 20 weeks’ gestation consistent with less severe NVP. By 34 weeks’ gestation, women with shNVP persisted in having lower weight gain compared with the no NVP group. Overall, there was a trend of lesser gestational weight gain with increasing severity of NVP up to 34 weeks’ gestation.

When we examined the potential association between NVP severity experienced earlier in the pregnancy with the later pregnancy outcomes of gestational diabetes, hypertensive disorders of pregnancy, induction of labour or caesarean delivery (elective and emergency), there were no associations found. However, with increasing NVP, there was a trend of decreasing gestational age at delivery (Table 2), which was small (half a week) but statistically significant in the shNVP group [β = − 0.51 weeks (− 0.91, − 0.11)] compared with the no NVP group. When the incidence of preterm delivery was considered, women with sNVP had increased odds of late preterm delivery (spontaneous and iatrogenic) after adjusting for covariates [adjusted odds ratio (aOR) 3.04 (95%CI 1.39, 6.68)]. Interestingly, this was not associated with an increased odds of NNU admission but a slightly lower odds of preterm NNU admission compared with the no NVP group. Birthweight centiles, and odds of SGA were unchanged with severity of NVP (Table 2), but there was an increased odds of LGA [aOR 1.54 (95%CI 1.00, 2.37)] in the mNVP group. The interaction terms of sex and ethnicity with NVP severity were not statistically significant for their association with gestational age or birthweight outcomes in our models.

**Table 2:** Pregnancy and neonatal outcomes

	**No vomiting**		**Mild-Moderate vomiting**		**Severe vomiting**		**Severe vomiting with hospitalisation**	
***Pregnancy Outcomes***	**% affected**	**aOR**	**% affected**	**aOR (95%CI)**	**% affected**	**aOR (95%CI)**	**% affected**	**aOR (95%CI)**
Gestational Diabetes	17.0	Ref	19.0	1.20 (0.83, 1.75)	21.7	1.28 (0.73, 2.23)	13.6	0.77 (0.34, 1.76)
Hypertensive Disorders	7.8	Ref	5.4	0.75 (0.42, 1.32)	5.7	0.77 (0.30, 1.99)	7.5	1.45 (0.51, 4.13)
Caesarean delivery	28.4	Ref	28.9	1.07 (0.78, 1.47)	33.3	1.33 (0.83, 2.13)	31.3	1.00 (0.53, 1.88)
Induction of labour	32.1	Ref	31.5	0.98 (0.71, 1.33)	29.3	0.93 (0.57, 1.52)	31.3	1.15 (0.62, 2.16)
***Timing of delivery***								
**Gestational age at delivery, weeks**	**median (IQR)**	**β**	**median (IQR)**	**β (95% CI)**	**median (IQR)**	**β (95% CI)**	**median (IQR)**	**β (95% CI)**
	39.0 (38.0, 39.0)	Ref	39.0 (38.0, 39.0)	−0.07 (−0.27, 0.12)	38.0 (37.0, 39.0)	−0.27 (− 0.58, 0.03)	38.0 (37.0, 39.0)	− 0.51 (− 0.91, − 0.11)*
**Preterm Delivery**	**% affected**	**aOR**	**% affected**	**aOR (95%CI)**	**% affected**	**aOR (95%CI)**	**% affected**	**aOR (95%CI)**
Early (< 34^+ 0^ weeks)	1.0	Ref	1.0	1.06 (0.27, 4.19)	0.0	–	1.5	–
Late (34^+ 0^–36^+ 6^ weeks)	4.8	Ref	5.5	1.14 (0.59, 2.19)	12.6	3.04 (1.39, 6.68)*	9.0	2.04 (0.69, 6.07)
***Neonatal Outcomes***								
**Birthweight centiles**	**median (IQR)**	**β**	**median (IQR)**	**β (95% CI)**	**median (IQR)**	**β (95% CI)**	**median (IQR)**	**β (95% CI)**
	53.5 (27.9, 75.5)	Ref	53.9 (26.7, 83.1)	−0.02 (−4.28, 4.23)	54.2 (31.1, 78.5)	1.71 (−4.86, 8.29)	51.3 (22.2, 80.7)	−0.60 (−9.15, 7.94)
	**% affected**	**aOR**	**% affected**	**aOR (95%CI)**	**% affected**	**aOR (95%CI)**	**% affected**	**aOR (95%CI)**
Small-for-gestational age	11.8	Ref	9.9	0.87 (0.55, 1.35)	8.1	0.71 (0.33, 1.51)	11.9	0.77 (0.30, 1.97)
Large-for-gestational age	10.8	Ref	17.3	1.54 (1.00, 2.37)*	13.8	1.32 (0.69, 2.52)	17.9	1.64 (0.74, 3.62)
**NNU admissions (all)**	4.8	Ref	3.4	0.66 (0.32, 1.34)	1.7	0.32 (0.07, 1.46)	11.9	1.29 (0.39, 4.19)
NNU admissions (preterm, < 37^+ 0^ weeks)	2.2	Ref	2.1	0.57 (0.16, 1.94)	0.7	0.07 (0.008, 0.80)*	5.2	0.97 (0.10, 9.27)
NNU admissions (term)	2.6	Ref	1.3	0.44 (0.14, 1.36)	1.0	0.35 (0.04, 3.02)	6.7	0.64 (0.07, 5.58)

All **β** and OR adjusted for ethnicity, maternal education, maternal age, parity, pre-pregnancy BMI, maternal smoking and offspring sex in multinomial logistic or linear regression. *p < 0.05

*Abbreviations*: *aOR* adjusted odds ratio, *IQR* interquartile range, *NNU* neonatal unit

**Fig. 1:**
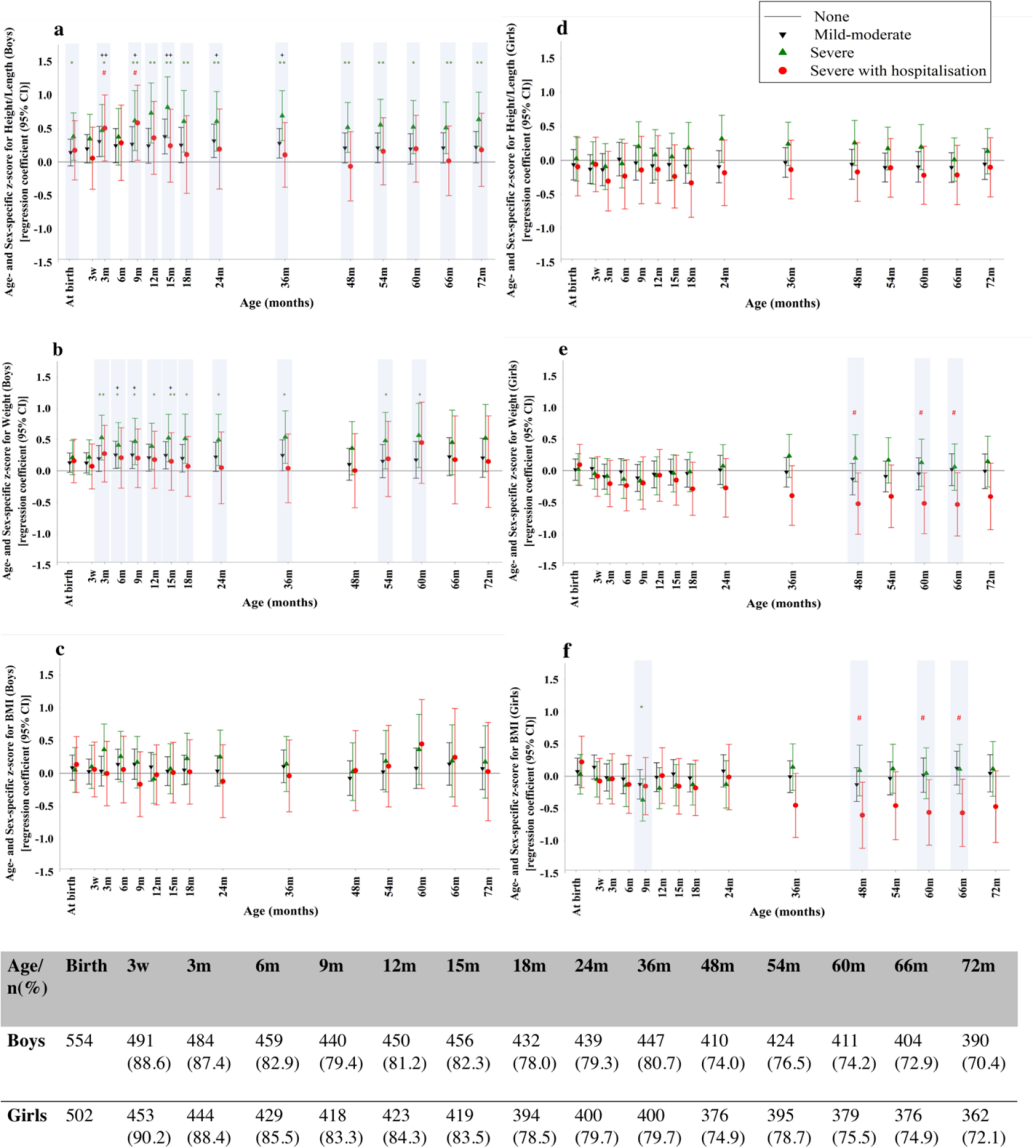
Association between severity of nausea and vomiting of pregnancy (NVP) and offspring anthropometry from birth to 72 months (m) in boys (**1a**-**1c**) and girls (**1d**-**1f**). The regression co-efficient in z-score for offspring length/height (**1a** and **1d**), weight (**1b** and **1e**) and body mass index (**1c** and **1f**) with 95% confidence intervals (CI) for each NVP group (mild-moderate: inverted black triangle; severe: green triangle; severe with hospitalisation: red dot) at each time point, relative to the reference group (no NVP, represented by the horizontal black line at 0 on the y-axis) is shown. Adjustment was made for maternal age, ethnicity, pre-pregnancy BMI, maternal education, parity, gestational age and smoking during pregnancy. Number of children contributing data at each time point is shown in the table. Statistically significant differences are highlighted by a light grey background with *p* values indicated as follows: No NVP vs mild-moderate: + *p* < 0.05, ++ *p* < 0.01; no NVP vs severe: * *p* < 0.05, ** *p* < 0.01; no NVP vs severe with hospitalization: # *p* < 0.05, ## *p* < 0.01

**Fig. 2:**
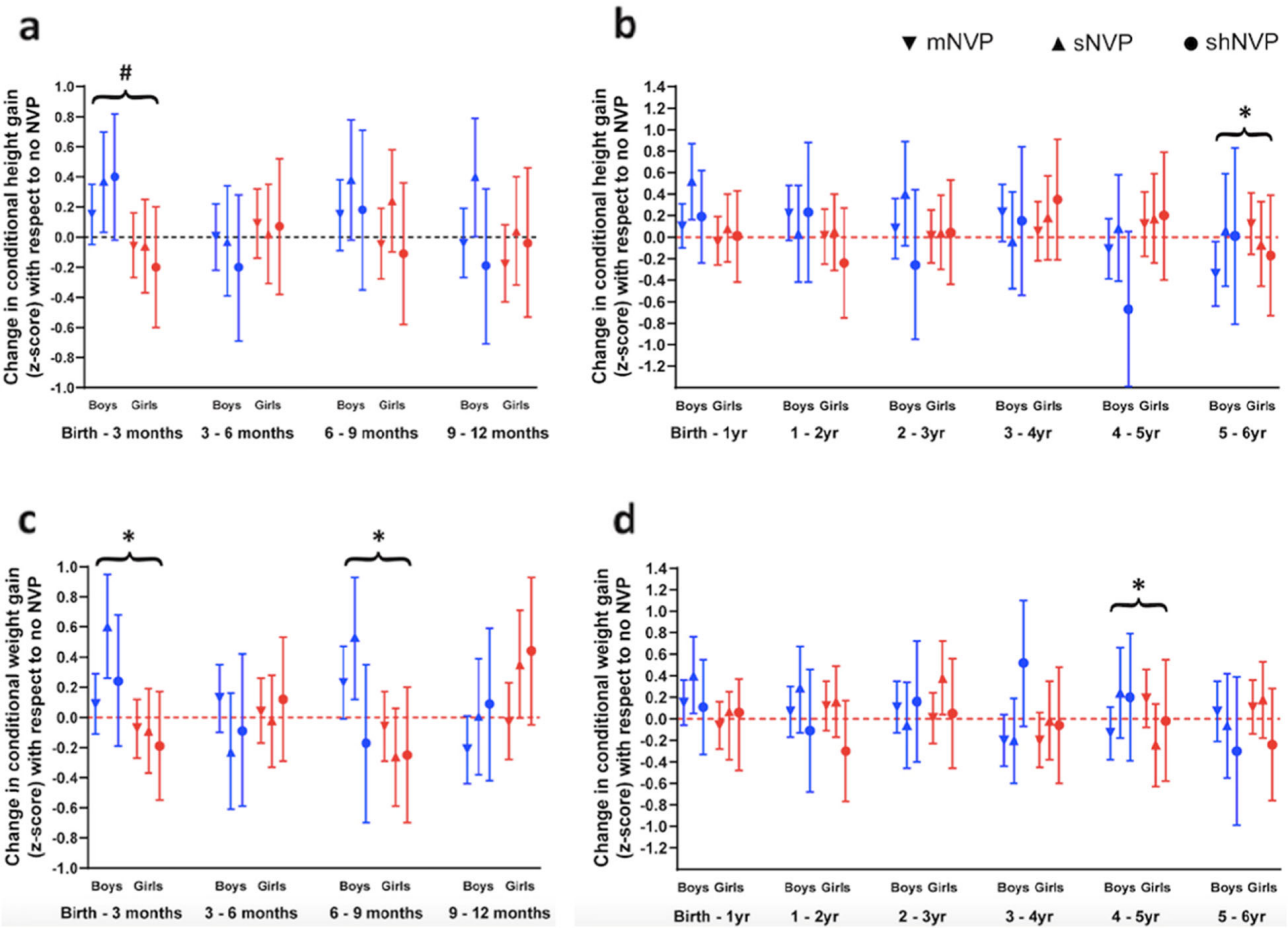
Association between the severity of nausea and vomiting of pregnancy (NVP) and change in offspring conditional length/height (**2a** and **2c**) or weight (**2b** and **2d**) gain in boys (blue) and girls (red), at 3-monthly intervals from birth to 12 months (**2a** and **2b**) and at annual intervals from birth to 72 months (**2c** and **2d**). The regression co-efficient in z-score with 95% confidence intervals (CI) for each NVP group [mild-moderate (mNVP): inverted triangle; severe (sNVP): triangle; severe with hospitalization (shNVP): dot] at each time point, relative to the reference group (no NVP, represented by the horizontal dotted line at 0 on the y-axis) is shown. Adjustment was made for maternal age, ethnicity, pre-pregnancy BMI, maternal education, parity and smoking during pregnancy. Statistically significant differences compared with the no NVP group occur where the 95%CI does not cross 0. Statistically significant interaction-p for sex differences are highlighted in brackets: *p < 0.05 and trend ^#^*p* = 0.101

Next, the association of NVP severity with childhood auxology was examined in boys and girls separately. Despite no significant differences in median birthweight centiles between the four NVP groups, other childhood anthropometry measures revealed sex-dependent differences (Figs. 1 and 2). Boys born to women with sNVP showed anthropometric differences from birth. They were longer at birth [adjusted difference in regression coefficients in the z-score (adjusted β) 0.38 standard deviations (SDs) (95% CI 0.02, 0.73)] and remained persistently taller in early childhood [adjusted β at 72 m 0.64 SDs (0.23, 1.04)] compared with the no NVP group (Fig. 1a). These boys became heavier from 3 m [0.53 SDs (0.18, 0.89)] up to 60 m [0.57 SDs (0.05, 1.08)] (Fig. 1b). The magnitude of differences (β) in height and weight z-scores between the sNVP and no NVP groups remained similar throughout early childhood. Even boys of mothers who only experienced mNVP showed significantly increased length/height between 3 m–36 m [adjusted β at 15 m 0.37 SDs (0.12, 0.61)] and increased weight between 6 m–15 m [adjusted β at 15 m 0.24 SDs (0.03, 0.45)], although to a lesser extent than the sNVP group, when compared to the no NVP group (Fig. 1a and b). Increments in weight were proportionate to height, so BMI was not significantly different between NVP groups throughout childhood (Fig. 1c).

To assess the time period when growth was most influenced by NVP, conditional height and weight gain were analysed (Fig. 2). Boys born to mothers with sNVP, grew faster than expected in the first 12 months of life. Compared with the no NVP group, boys from the sNVP group demonstrated increased conditional length gain [adjusted β 0.52 SDs (0.16, 0.87)] and weight gain [0.40 SDs (0.05, 0.76)] at birth-12 m (Fig. 2b, d). This gain in length and weight was especially marked during the intervals of birth-3 m [0.37 SDs (0.03, 0.70), and 0.60 SDs (0.26, 0.95), respectively] and 6-9 m [0.38 SDs (− 0.02, 0.78), and 0.53 SDs (0.12, 0.93), respectively] (Fig. 2a, c).

Conversely, in girls born to mothers with any degree of NVP, length/height remained similar across childhood (Fig. 1d). However, girls born to mothers who experienced shNVP began to show lower weight from 48 m [− 0.52 SDs (− 1.00, − 0.03)] and remained so at 66 m [− 0.53 SDs (− 1.03, − 0.03)] (Fig. 1e), accompanied by a similarly lower BMI at 48 m [− 0.61 SDs (− 1.12, − 0.09)] and 66 m [− 0.57 SDs (− 1.09, − 0.05)] compared to the no NVP group (Fig. 1f). Conditional height/weight gain analyses (Fig. 2) did not reveal significant growth reductions with any degree of NVP compared with no NVP. Of note, compared with boys, girls born to mothers with NVP were slower to gain weight, particularly at birth-3 m (sex-interaction *p* = 0.028) and 6-9 m (sex-interaction *p* = 0.021), and showed a trend of slower length gain at birth-3 m (sex-interaction *p* = 0.101) (Fig. 2a and c). Further sex differences in growth patterns was observed at 4–5 years, with weight gain appearing slower in girls than boys from the sNVP group.

## Discussion

Overall, 75% of women in this multi-ethnic Asian cohort demonstrated some NVP, with 10% having sNVP and 5.8% shNVP. A novel finding is that Malay and Indian women were at higher risk of experiencing sNVP and shNVP compared with Chinese women. Consistent with other studies, we found that severe NVP was associated with carriage of a girl and a higher risk of late preterm birth. We also discovered sex-dependent associations between NVP and offspring growth with boys being larger-sized from birth onwards, gaining weight and height faster than expected in the first year of life. Whereas, girls were lighter with lower BMI in early childhood.

In published studies, being of Asian descent is a recognised risk factor for NVP [23] and higher rates of severe NVP of up to 10% have been reported in studies of Chinese and Japanese women [23, 24], similar to the incidence of sNVP reported in this study. However, definitions of NVP severity differ between studies, with shNVP being the most severe end of the spectrum within this cohort, so direct comparisons between the NVP rates reported here with other populations is imprecise.

Malay and Indian women might be more vulnerable to NVP than the Chinese due to genetic, endocrine and dietary differences. No studies have yet investigated differences in these aspects between different Asian ethnicities with most studies combining data of all Asian women. From the genetic perspective, among European women, variants in placenta and appetite genes such as GDF15 and IGFBP7 have been associated with hyperemesis gravidarum [23, 24] as has circulating levels of GDF15 [25]. One postulation could be that polymorphisms of specific genes may partly explain varying risks of severe NVP among Asian ethnicities. Another proposed theory is that pregnant Asian women have a greater elevation of human chorionic gonadotrophin concentrations associated with suppression of thyroid stimulating hormone [26] compared to other populations, which may influence NVP severity. This theory is supported by the observation that women with multiple and molar pregnancies demonstrate such biochemical changes and commonly experience worse NVP [27–29]. Asian populations have a higher prevalence of *H. pylori*, which has also been thought to play a role in NVP [30, 31]. There are also variations in cultural and societal expectations of behaviours during pregnancy that may influence the perception and experience of NVP [32].

Despite babies being born slightly earlier in the sNVP group, consistent with published meta-analyses of increased preterm delivery associated with hyperemesis gravidarum [3], there were no increased odds of admission to NNU as delivery occurred predominantly after 34 weeks’ gestation when the likelihood of NNU admission is low. It is known that poor gestational weight gain, malnutrition, and low BMI in pregnant women have been associated with preterm delivery [4]; similar factors could be mediating the effect of shorter gestation at birth with increasing severity of NVP. The absence of an increased odds of preterm delivery in the shNVP group may be due to the smaller sample size and lower statistical power.

Interestingly, sex-dependent differences in growth were observed in offspring born to mothers with NVP, even at milder severity. Boys born to women with mNVP and sNVP were already on the larger side at birth, grew faster in infancy and remained larger in early childhood. The presence of NVP could be a reflection of placental hormonal secretion and hence general placental health, promoting fetal growth [4], particularly in males, who may be more sensitive to differences in the hormonal milieu during pregnancy given their greater intrauterine growth velocity [33]. Such a concept would also be in line with findings that NVP was associated with lower miscarriage rates, suggesting that NVP may be linked with improved quality of implantation and placentation even in early pregnancy [34].

Conversely, girls born to mothers with shNVP showed lower weight and BMI from 48 to 66 m [35]. We speculate that in girls, fetal exposure to severe NVP may change *in utero* metabolic programming due to alterations in maternal hormones and nutritional supply [35]. In line with this idea, studies of girls born to GDM mothers had shown poorer weight gain in childhood, despite presumably increased transplacental glucose supply pre-GDM-diagnosis followed by a relative reduction following GDM treatment, a phenomenon not observed in boys [12, 36, 37]. Thus the apparent direction of change in fetal nutritional supply and the timing at which the change occurs may be associated with some counter-intuitive effects on later child growth. Other NVP-associated endocrine disturbances such as higher levels of leptin [38], increased oxidative stress [39, 40], not measured here, may also be implicated in the susceptibility of girls to altered metabolic programming.

The pathophysiological mechanisms underlying these sex-dependent childhood growth differences are unknown. In addition, whether these outcomes persist beyond 72 m and have longer-term implications on health of these children is unclear. The Dutch famine study has shown that depending on the timing of malnutrition, effects on birthweight differ, and regardless of the initial birthweight, adverse long-term effects on cardiometabolic health can occur [35]. Given the alterations in early childhood auxology observed with NVP in our study, even at milder forms, it is imperative that further research is conducted to assess if there are related changes in body composition and metabolism, and indeed longer-term implications in later life.

This is a prospective, longitudinal study where mothers’ recollection of earlier NVP was gathered in mid-pregnancy before any study outcomes had manifested. However, there could be recall bias in their self-reported NVP symptoms. Due to the nature of our data collection that was based on a questionnaire with incomplete details in hospital records, it is uncertain if those with shNVP would have met the hyperemesis gravidarum criteria. Also, the shNVP group lacked statistical power as it was limited by small sample size. Even though lost to follow-up of offspring across the four NVP groups between birth and 72 m was approximately 30%, it was similar across NVP groups. Hence, this would cause little bias in child outcomes. Residual confounding, such as maternal dietary intake and mental health, that have not been adjusted for in the analyses could also exist.

## Conclusions

In conclusion, severe NVP was more likely in Malay and Indian women than the Chinese, and was associated with an increased odds of late preterm delivery. Boys born to women with NVP were larger and longer at birth, and showed greater weight and height gain in infancy. Conversely, girls were lighter with reduced BMI in early childhood. These findings will need verification in separate mother-offspring cohorts, and in particular, generalisability to non-Asian populations needs to be explored. As even milder severities of NVP could be associated with long-term alterations in offspring growth, further research is needed to determine possible underlying mechanisms involved and implications on future offspring health.

## Data Availability

The datasets used and/or analysed during the current study can be made available from the corresponding author upon approval of an application to the GUSTO executive committee.

## Abbreviations

NVP: Nausea and vomiting of pregnancy
mNVP: Mild to moderate nausea and vomiting of pregnancy
sNVP: Severe nausea and vomiting of pregnancy
shNVP: Severe nausea and vomiting of pregnancy with hospitalisation
GUSTO: Growing Up in Singapore Towards healthy Outcomes
GDM: Gestational diabetes mellitus
GWG: Gestational weight gain
BMI: Body mass index
NNU: Neonatal unit
SGA: Small for gestational age
LGA: Large for gestational age
